# Advancing micro-electrometric techniques for the detection of organophosphate and carbamate residues using cricket cholinesterase

**DOI:** 10.1371/journal.pone.0308112

**Published:** 2024-07-31

**Authors:** Anurak Wongta, Priyanshi Anand, Nealler A. A. Aning, Nootchakarn Sawarng, Surat Hongsibsong

**Affiliations:** 1 Research Institute for Health Sciences, School of Health Science Research, Chiang Mai University, Chiang Mai, Thailand; 2 Research Institute for Health Sciences, Environmental and Occupational Health Sciences Unit, Chiang Mai University, Chiang Mai, Thailand; 3 Faculty of Science, Asia-Pacific International University, Saraburi, Thailand; 4 Faculty of Medicine, Department of Community Medicine, Chiang Mai University, Chiang Mai, Thailand; University of Nebraska Medical Center, UNITED STATES OF AMERICA

## Abstract

The widespread use of organophosphate (OP) and carbamate (CM) pesticides requires efficient and cost-effective detection methods. This study introduces a micro-electrometric method using cricket cholinesterase (ChE) to detect OP and CM residues, providing a rapid and economical alternative to conventional chromatographic techniques. The parameters of the method, including the substrate concentration, incubation temperature, and incubation time, were optimized. By leveraging the sensitivity of cricket ChE to OP and CM inhibition, this approach translates enzyme inhibition into an electrical signal to quantify pesticide levels, achieving an impressive limit of detection (LOD) from 0.036 to 0.086 parts per million (ppm). This method demonstrated reproducibility and stability, making it suitable for field applications and on-site testing across various environmental matrices. This research represents a significant advancement in pesticide residue analysis with potential applications in the development of portable biosensor devices for real-time environmental monitoring and public health protection.

## Introduction

The widespread use of pesticides in agriculture and various industries has significantly improved crop yield and overall productivity. Nevertheless, this has raised increasing concerns regarding its potential negative impact on human health and the environment [1]. Pesticides, especially organophosphates (OP) and carbamates (CM) are used globally to control pests and increase agricultural output. However, these residues can remain in crops and pose serious health risks when consumed in large quantities. These pesticides inhibit acetylcholinesterase, an enzyme essential for normal nervous system function, and prolonged exposure to them can lead to neurological issues, and reproductive disorders [2,3]. Monitoring these residues is crucial to safeguard the health of consumers and agricultural workers.

Pesticide residues are traditionally detected using gas chromatography (GC) and high-performance liquid chromatography (HPLC), which are expensive, time-consuming, and require complex equipment that is not available in all laboratories [4–7]. Immunoassays offer high sensitivity and specificity but also require advanced equipment and skilled operation, often involving animal-derived antibodies [8,9]. Alternative techniques, such as optical colorimetric assays, fluorometric assays, electrochemical biosensors, rapid test cards, and microfluidic devices, utilize enzyme inhibition, specifically cholinesterase, to detect pesticides such as organophosphates and carbamates. A notable innovation includes a bioactive paper sensor embedded with substances such as chitosan, glutaraldehyde, acetylcholinesterase (AChE), and acetylthiocholine iodide (ATCh), which changes color in the presence of AChE inhibitors, providing a rapid and economical screening solution [10–15]. However, despite their robust detection capabilities and reproducibility, potentiometric OP biosensors face challenges such as the need for high potentials and fouling issues that affect their efficacy [16].

At cholinergic synapses, acetylcholinesterase effectively hydrolyzes the neurotransmitter acetylcholine into choline and acetic acid [17], playing a vital role in the detection of pesticide residues as it is inhibited by OP and CM pesticides [18]. Butyrylcholinesterase (BChE), another enzyme that originates in the liver, breaks down various substances, including acetylcholine and numerous drugs, although it is less efficient than AChE. BChE levels vary among individuals owing to genetic and health factors. Notably, BChE levels in human plasma decrease faster than AChE levels in red blood cells but rebound more rapidly after exposure to OP [19].

Insect cholinesterase rapidly breaks down acetylthiocholine. The majority of cholinesterase (ChE) activity in insects is concentrated in the central nervous system rather than in the peripheral nervous system. A distinct type of cholinesterase that processes acetylcholine and is blocked by physostigmine has been found in the muscle motor endplates of crickets. The application of insecticides has demonstrated that critical sites within the brain and nerve cords of crickets are crucial for knockdown [20]. Consequently, ChE sourced from crickets is a promising option for developing detection methods for pesticide contamination that inhibit neurotransmitters such as OPs and CMs.

Previous screening methods for pesticide contamination have certain limitations, including high costs, reliance on sophisticated equipment, and complexity. Therefore, a more comfortable and cost-effective method is required for pesticide residue detection. The potentiometric method allows rapid results, which are crucial for field applications. The simplicity of this method eliminates the need for complex laboratory equipment, enabling on-site testing and immediate decision making. A microelectrometric method for pH detection involves utilizing micro/nanosized pH sensors to monitor pH changes on a small scale, providing high spatial resolution measurements [21]. These sensors can be fabricated using materials such as carbon modified with polypyrrole nanotubes for enhanced sensitivity and detection of pH changes during processes such as loop-mediated isothermal amplification (LAMP) for virus diagnosis [22]. Such pH sensor systems can be crucial in various applications, including biological studies, corrosion science, energy applications, and environmental research, offering cost-effective and efficient means of pH monitoring and analysis. This approach is particularly beneficial in low-resource settings and significantly reduces testing costs by requiring smaller sample sizes and common equipment such as a portable pH meter.

This investigation focused on developing an in-house micro-electrometric method for identifying the residues of organophosphates and carbamates using cricket cholinesterase. This method is intended to be cost effective and easy to use.

## Materials and methods

### Cricket cholinesterase

The cholinesterase enzyme from house crickets, utilized in our previous research, was prepared using the following process: sample extraction was conducted by combining 1 mg of the sample with 8 ml of phosphate-buffered saline (PBS) buffer. Specifically, 5 mg of the sample was ground into a paste and diluted with 40 ml of 1% Triton X-100 in PBS at pH 7.2. After centrifugation at 10,000 g for 30 min at 4°C, 30 ml of the supernatant was collected. Proteins were then precipitated using a salting-out technique with ammonium sulfate, enhancing protein-protein interactions by increasing the salt concentration, which causes the proteins to aggregate and settle. To achieve this, 10 ml of the extract was mixed with a suitable volume of saturated ammonium sulfate, stirred at room temperature for an hour, and then centrifuged at 12,000 g for 30 min at 4°C. The supernatant was discarded, and the protein pellet was resuspended in 1 ml of PBS at pH 7.2 [23]. The protein solution was stored at -20°C until measurements of cholinesterase (ChE) activity were taken in a subsequent step.

### Chemicals

Standard pesticides, including dicrotophos, dichlorvos, mevinphos, carbaryl, carbosulfan, and methomyl were purchased from Dr. Ehrenstorfer GmbH (Augsburg, Germany). Acetylthiocholine iodide (ATCh) was purchased from Sigma Aldrich. Human recombinant acetylcholinesterase (AChE) was purchased from Sigma-Aldrich (USA)

### Apparatus

Desk pH meter (OHAUS, USA)

Portable pH meter (Horiba LAQUAtwin pH-33 Compact Meter Portable pH Meter, Japan)

### Electrometric procedure for measurement of ChE activity

In our study, we employed a revised electrometric technique developed by Mohammad et al. [21] to assess ChE activity levels. This method involved conducting an assay in a 10 ml beaker containing a mixture of 3 ml distilled water, 200 μl of ChE enzyme sample, and 3 ml of PBS which is composed of 137 mmol/l NaCl, 2.7 mmol/l KCl, 10 mmol/l Na₂HPO₄, and 1.8 mmol/l KH₂PO₄, totaling a phosphate concentration of 11.8 mmol/l. The pH was adjusted to 8.1 using NaOH. Initially, the pH of the mixture (denoted as pH1) was determined using a glass electrode connected to a pH meter (OHAUS, USA). Then, 0.1 ml of a 7.5% acetylthiocholine iodide stock solution was introduced into the beaker. The mixture was then incubated at a temperature of 37°C for a duration of 20 min. Upon completion of incubation, a second pH measurement (pH2) was performed. ChE activity was quantified by calculating the change in pH over 20 min, adjusted for the change observed in the control sample without ChE, using the following formula:

ChEactivityΔpHat20min=(pH1–pH2)−ΔpHofthecontrol.

### Optimization of micro-electrometric method for Cricket ChE activity

The precision of the portable pH meter and the conventional desktop pH meter was evaluated using the methods outlined in the "Electrometric procedure for measurement of ChE activities" using standard acetic acid solutions of different concentrations (0–15 mM). The results validated the suitability of employing a portable pH meter for subsequent phases of development.

To reduce the use of chemicals and samples in the assay to microliter volumes, the conventional procedure was adapted as follows: A volume of 20 μl of 0.2 U standard AChE where one unit of AChE activity is defined as the amount of enzyme needed to hydrolyze 1 μmol of ATCh per min at pH 8.0 at 37°C, was mixed with 300 μl of distilled water in a microcentrifuge tube, to which 300 μl of PBS at pH 8.1 was subsequently added. The initial pH (pH1) was measured using a portable pH meter. Subsequently, 10 μl 7.5% acetylthiocholine iodide solution was added to the mixture. This was incubated at 37°C for 20 min. After the incubation, a second pH measurement (pH2) was performed. The enzyme activity was determined using the same calculation method as that used for the standard protocol. The results were compared with those obtained using the standard method.

The microelectrometric method was applied to the cricket ChE, and the optimal concentration for the ATCh substrate was optimized. This involved conducting pH assessments with cricket ChE and varying concentrations of the ATCh substrate, ranging from 3.75% to 30% as stock solutions (4.0 mM to 32.3 mM, final concentration).

The temperature and time were optimized. As detailed in the previous section, the methodology involved evaluating the ChE activity at two distinct temperature settings and several time points. Measurements were conducted in a water bath set at 25°C and 37°C, respectively. Enzyme activity was quantified at intervals of 5 min from the start to 30 min, using the same computational approach outlined in the standard protocol. A comparison of the outcomes led to the selection of temperature conditions that demonstrated superior activity levels for use in subsequent stages of the study.

The effect of methyl alcohol (MeOH) on ChE activity was examined using seven different concentrations of MeOH in PBS (0–5% v/v) as a buffer solution for ChE activity assays. Upon comparing the results of these assays, the specific MeOH concentration did not influence ChE activity and was subsequently employed to extract pesticides from vegetable samples.

### Optimization of the method for ChE inhibition test

AChE plays a pivotal role in the nervous system by breaking down the neurotransmitter acetylcholine, which is essential for seamless communication between the nerve cells. The reaction facilitated by cholinesterase typically converts acetylcholine (C7H_16_NO_2_^+^) to acetic acid (CH_3_COOH) and choline (C_5_H_14_NO^+^), as illustrated by the following chemical reaction:

$$\mathrm{Acetylcholine}(C_{7}H_{16}{\mathrm{NO}}_{2}+)\;\overset{\mathrm{AChE}}{\to }\;\mathrm{Acetic}\;\mathrm{acid}({\mathrm{CH}}_{3}\mathrm{COOH})+\mathrm{choline}(C_{5}H_{14}\mathrm{NO}+).$$

Electrometric assays effectively detect shifts in cholinesterase activity, which is a key indicator of inhibition by organophosphates and carbamates. These assays use a substrate that changes the pH when interacting with cholinesterase, which typically breaks down the substrate and alters the pH of the solution. This pH change was reduced or absent when cholinesterase was inhibited, allowing for the determination of the presence of pesticides.

Two dilutions of cricket ChE (1:1–1:2 with final concentrations of 0.58 and 0.29 mg/ml) were used as samples and two concentrations of stock ATCh (15% and 30% with final concentrations of 16.1 mM and 32.3 mM) were used as substrates. The ΔpH value of each reaction was measured using the optimized conditions from the previous section, “Optimization of Micro-Electrometric Method for Cricket ChE Activity,” which was performed in a total volume of 630 μL with a reaction time of 20 min at 25°C. A combination of diluted ChE and concentrated ATCh with the best ΔpH at 20 min was selected to further develop the ChE inhibition test.

The procedure to determine the ideal duration for an inhibition assay was carried out as follows: 20 μl of the cricket ChE enzyme was introduced into a tube, followed by the addition of 300 μl of mevinphos at a concentration of 1 part per million (ppm) in 5% methyl alcohol (MeOH) PBS at pH 8.1, acting as the inhibitor. This mixture was then incubated for five durations ranging from 10 to 25 min. Subsequently, 300 μl distilled water (DW) was added. The ChE activity was evaluated at room temperature (25°C) at 20 min as described in the previous section. From these measurements, the percentage inhibition was calculated to determine the most suitable incubation time for subsequent use in assessing ChE inhibition by OP and CM pesticides. The inhibition rate was calculated using the following equation:

%Inhibition=(ΔpH0‐ΔpH)/ΔpH0×100

where [ΔpH0] is the initial enzyme activity in the absence of an inhibitor and [ΔpH] is the enzyme activity in the presence of an inhibitor.

### ChE inhibition by organophosphate and carbamate pesticides

In this assay, 20 μl of 1:2 diluted cricket ChE was dispensed into a tube, followed by the introduction of 300 μl of OP and CM inhibitors at varying concentrations ranging from 0.008 to 5.000 ppm, dissolved in 5% MeOH PBS with a pH of 8.1. The solution was incubated for 20 min, after which 300 μl DW was added. The activity of the ChE enzyme was assessed at a standard room temperature of 25°C, following the methodology outlined in the previous section. A calibration curve was plotted, from which the half-maximal inhibitory concentration (IC_50_) and the concentration causing 25% inhibition (IC_25_) were determined using the Prism software version 4.01. The IC_25_ value was designated as the detection limit (LOD) for this analytical method.

### Extraction of pesticides from vegetable samples

The extraction procedure was modified from a previous study [23]. For each vegetable sample, 5 g was chopped finely and shaken with 10 ml of dichloromethane for 5 min. Subsequently, 5 ml of the extract was decanted into a glass tube and evaporated in a hot water bath until the solvent was completely dissipated. The residue was reconstituted in 300 μl dichloromethane, followed by the addition of 300 μl of 5% MeOH PBS at pH 8.1, and the mixture was thoroughly shaken. The dichloromethane was removed by heating in a hot water bath. The resultant solution was used to assess the ChE enzyme activity in the subsequent phase.

### ChE inhibition by organophosphate and carbamate pesticides in vegetables

Pesticide-free cabbage, previously verified to be devoid of OP residues by gas chromatography with a flame photometric detector (GC-FPD), as reported in a previous study [24], was artificially contaminated with various concentrations (0.008–5.000 ppm) of several pesticides, including dicrotophos, dichlorvos, mevinphos, carbaryl, carbosulfan, and methomyl. Following the extraction protocol outlined in the previous section, the prepared samples were analyzed using the developed method to assess inhibition levels. Briefly, 20 μl of 1:2 diluted cricket ChE (final concentration 0.29 mg/ml) was placed in a tube and 300 μL of the sample extract was added. This mixture was incubated for 20 min and then diluted with 300 μL distilled water. The pH of each mixture was measured to determine ChE activity using a previously described calculation for inhibition. A calibration curve was constructed to determine IC_50_ and IC_25_ using Prism software version 4.01. The IC_25_ value was designated as the LOD.

### Application of the developed method to vegetable samples

The 33 vegetable samples were analyzed via GC-FPD, as described in our previous study [24], including a variety of species, such as kale (*Brassica alboglabra L*.*H*. *Bailey*), cabbage (*Brassica oleracea var*. *capitata L*.) and long beans (*Vigna unguiculata ssp*. *Sesquipedalis*), and morning glory (*Ipomoea aquatica Forssk*.). Following a previously described extraction method, the samples were subjected to the developed method to assess their levels of inhibition. The calculation of inhibition was performed according to a previously outlined methodology. The samples that exhibited inhibition exceeding 25% were interpreted as insecticide residues. To evaluate the relative sensitivity, specificity, accuracy, positive predictive value, and negative predictive value of the assay, the outcomes were quantified and compared with those obtained from GC-FPD [25].

### Statistical analysis

The results of ChE activity are presented as the average and standard deviation. The inhibition curve was plotted, and the IC_50_ was determined using GraphPad Prism 4.01. Differences in ChE activity and inhibition percentages throughout the study were evaluated using the t-test and One-way ANOVA. All statistical tests were two-sided with a significance threshold of 0.05. SPSS version 17 was used for all analyses.

## Results

**Fig 1:**
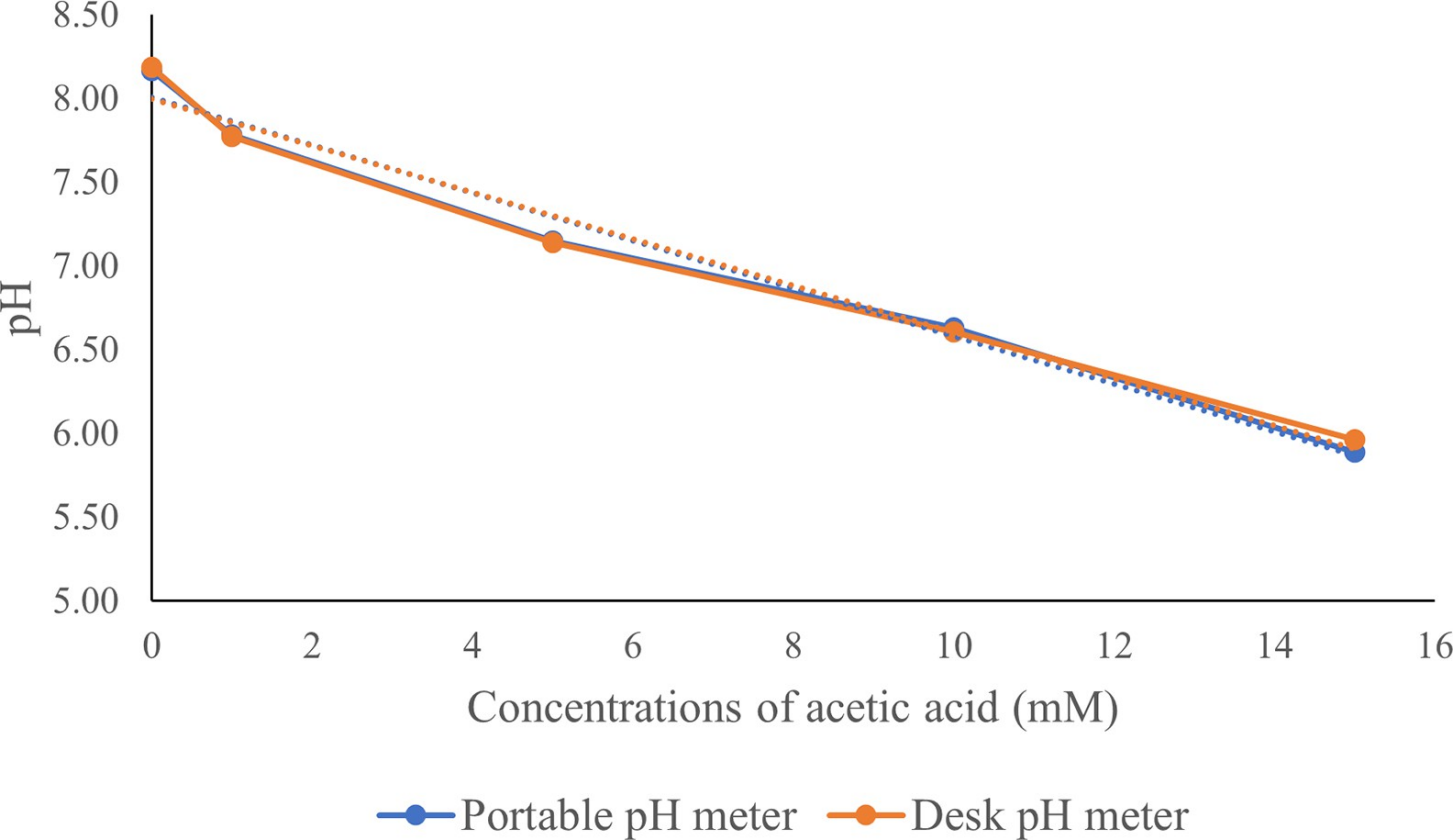
Comparison of pH values at different concentrations (0–15 mM) of standard acetic acid in 11.8 mmol/L PBS (pH 8.1), measured using two pH meters.

The test results, comparing the accuracy of a standard desk pH meter and a portable pH meter using a standard acetic acid solution at various concentrations (0–15 mM), showed similar values, as shown in Fig 1.

The results of the experiment were reduced from milliliters to microliters. By reducing the overall volume from the Muhamadd method (6.3 ml) to a developing method with an overall volume of only 630 μl under the same experimental conditions, using samples of 0.2 U standard AChE and ATCh as substrate, as well as PBS at pH 8.1, as a buffer solution in the test method, similar ΔpH at 20 min values were found. Instead, the developed microelectrometric method was found to have more significant value variability, as shown in Table 1, and no difference was demonstrated between the methods.

**Table 1:** Comparison of cholinesterase activity (ΔpH) obtained from the original and developing micro methods using a 0.2 U standard AChE sample.

Method	Original method	Developing micro method	*p*-Value
**ΔpH at 20 min (mean±SD)**	0.26±0.01^a^	0.27±0.02^a^	0.597

Means followed by the same letter are not significantly different (P > 0.05, Independent Sample T-Test).

Applying the microelectrometric method to cricket ChE and the subsequent optimization of the ATCh substrate concentration revealed significant findings. As indicated in Table 2, the examination of ChE activity across four levels of stock ATCh substrate concentrations (ranging from 3.75% to 30% w/v, with final concentrations from 4.0 mM to 32.3 mM) demonstrated a progressive increase in ΔpH, a measure of enzymatic activity. The data showed that the 30% stock ATCh substrate concentration yielded the highest ΔpH change at 20 min, with a mean of 0.25±0.01, compared to the lower stock concentrations of 3.75%, 7.5%, and 15%, which resulted in ΔpH means of 0.02±0.01, 0.06±0.01, and 0.12±0.01, respectively. This optimized concentration is pivotal for accurately evaluating the inhibitory effects of ChE, particularly in pesticide exposure studies.

**Table 2:** The result of cholinesterase activity (ΔpH) in 4 levels of stock acetylthiocholine iodide (3.75% to 30% w/v, with final concentrations from 4.0 mM to 32.3 mM).

	Stock ATCh concentration (%)			
	3.75%	7.5%	15%	30%
**ΔpH at 20 min (mean±SD)**	0.02±0.01	0.06±0.01	0.12±0.01	0.25±0.01

Abbreviations: ΔpH; pH1 -pH2, ATCh; Acetylthiocholine iodide; Means followed by the same letter are not significantly different (P > 0.05; One-Way ANOVA; post-hoc Bonferroni multiple comparisons).

**Fig 2:**
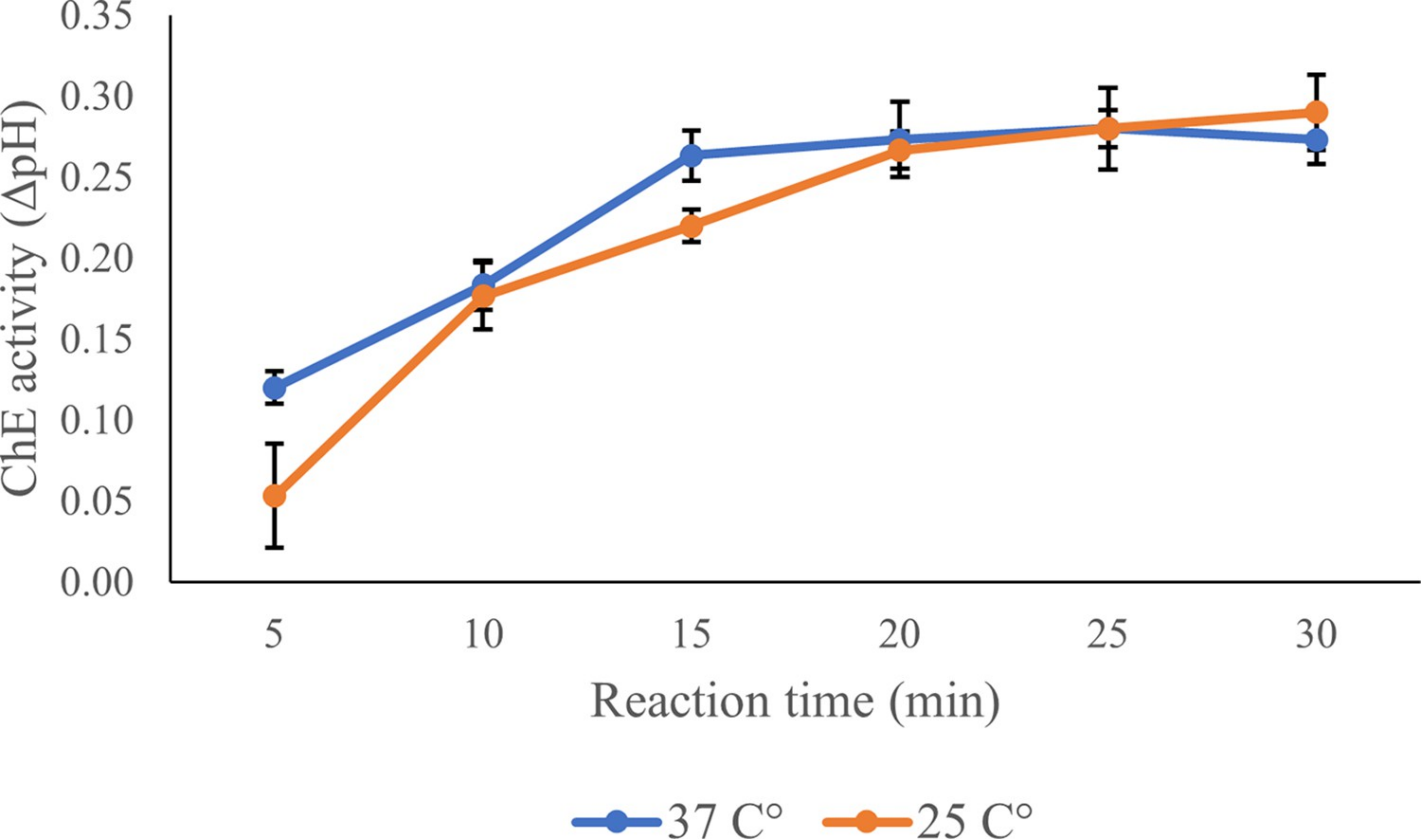
Comparative analysis of cricket ChE activity over time at 25°C and 37°C.

Upon optimizing the assay conditions for ChE activity, it was found that while the enzyme exhibited increased activity at 37°C, there was a notable intersection in activity levels between two temperatures at the 20-minute mark, as shown in Fig 2. The enzyme activities at 25°C and 37°C were comparable at this point. Considering the practical aspects of assay deployment, particularly for on-site applications, the ease of maintaining a consistent temperature of 25°C without specialized equipment makes it a more viable option for fieldwork. Therefore, despite the slightly higher activity observed at 37°C in later stages, 25°C was chosen for further steps in the methodology because of its practicality and sufficient enzyme activity observed at the 20-minute interval. This decision underscores the importance of balancing optimal laboratory conditions with the feasibility of real-world applications.

### The optimal condition for Cholinesterase inhibition

We assessed the effects of various concentrations of MeOH, ranging from 0% to 5% v/v, in a phosphate buffer solution with a pH of 8.1 on ChE activity. The results in Table 3 reveal no significant differences in the ChE activity across the tested MeOH concentrations. This uniformity in enzyme performance indicates that a 5% MeOH concentration in PBS at pH 8.1 is adequate for resuspending extracted samples without altering enzyme activity. Consequently, 5% MeOH in PBS was used for sample preparation in subsequent assays.

**Table 3:** The result of cricket ChE activity in 6 levels (%V/V) of MeOH in PBS pH 8.1.

	Concentration of MeOH in PBS (%)					
	5	4	3	2	1	0
**ΔpH at 20 min (mean±SD)**	0.26±0.03^a^	0.27±0.02^a^	0.25±0.01^a^	0.25±0.01^a^	0.25±0.01^a^	0.27±0.00^a^

Abbreviations: ΔpH; pH1 -pH2,; MeOH, methanol; PBS, phosphate-buffered saline. Means followed by the same letter are not significantly different (P > 0.05; One-Way ANOVA; post-hoc Bonferroni multiple comparisons).

To identify the optimal dilution ratio for cricket ChE and the appropriate concentration of the ATCh substrate for the inhibition assay, various combinations were evaluated using the developed ChE activity method. The findings showed that both undiluted ChE and a 1:2 dilution, when paired with 30% stock ATCh substrate concentration, produced identical results, yielding a ΔpH of 0.21 at the 20-minute mark, as outlined in Table 4. Consequently, a mixture of 1:2 diluted ChE (final concentration 0.29 mg/ml) and 30% stock ATCh (final concentration 32.3 mM) was chosen for the subsequent stage of inhibition testing.

**Table 4:** Comparative analysis of cricket ChE Activity under variable dilutions and stock ATCh Concentrations.

	ΔpH at 20 min (Mean±SD)	
	Dilution (1:x)	
**Stock ATCh (%)**	**1**	**2**
15	0.19±0.01	0.16±0.01
30	0.21±0.00	0.21±0.00

Abbreviations: ΔpH; pH1 -pH2, ATCh; Acetylthiocholine.

Additionally, to refine the inhibition assay, the time required for complete interaction between the pesticides and ChE was determined. It was ascertained that 20 min was adequate for the complete inhibition of ChE activity by 1 ppm mevinphos, with no noteworthy differences detected after this interval, as shown in Table 5.

**Table 5:** Kinetic profiling of cricket cholinesterase inhibition by mevinphos at various incubation intervals.

	Incubation time (min)				
	5	10	15	20	25
**% Inhibition (Mean±SD)**	67.8±1.91	84.47±1.96^a,b^	88.9±3.3^a,b,c^	92.47±3.37^a,b,c^	93.37±3.87^b,c^

Abbreviations: Means followed by the same letter are not significantly different (P > 0.05; one-way ANOVA; post-hoc Bonferroni Multiple Comparisons).

### Development and application of the ChE inhibition method for vegetable samples

Utilizing the optimized ChE inhibition approach, this study determined the IC_50_ and IC_25_ values of OP and CM pesticides. IC_25_ was used as the limit of detection (LOD) for this method. The LOD values ranged from 0.029–0.063 ppm for standard OPs and–0.036–0.071 ppm for fortified samples. For the standard CM pesticides and fortified samples, the LODs were–0.011–0.086 ppm and 0.061–0.086 ppm, respectively. As shown in Table 6, among the OP pesticides, mevinphos was the most potent inhibitor, and among the CM pesticides, carbosulfan was the most potent.

**Table 6:** Detection limits of standard pesticides and fortified samples using the cricket ChE inhibition method.

	Detection limits (ppm)	
Pesticides	Standard pesticides	Fortified samples
**Dicrotophos**	0.063	0.071
**Dichlorvos**	0.055	0.036
**Mevinphos**	0.029	0.071
**Carbarly**	0.086	0.086
**Carbosulfan**	0.05	0.061
**Methomyl**	0.011	0.084

Abbreviations: ppm; part per million.

According to the data in Table 7, evaluating the effectiveness of the developed ChE inhibition method against the GC-FPD method in 33 vegetable samples resulted in 21 true positives, 9 true negatives, 1 false positive, and 2 false negatives. This led to a relative sensitivity of 91.3%, relative specificity of 90%, accuracy of 91%, positive predictive value of 96%, and negative predictive value of 82%.

**Table 7:** The efficiency of the developed cricket ChE inhibition method for the detection of pesticides in vegetable samples compared with GC-FPD.

	Number of positive samples (n)		Number of negative samples (n)	
Methods	TRUE	FALSE	TRUE	FALSE
**GC-FPD**	23	0	10	0
**ChE inhibition method**	21	2	9	1

## Discussion

The findings of this study demonstrate the efficacy of an in-house developed microelectrometric method utilizing cricket cholinesterase for detecting OP and CM pesticide residues. This novel approach addresses several limitations of traditional detection methods, such as GC and HPLC, which are often costly and complex [4–7]. The developed method provides a rapid and cost-effective alternative suitable for on-site testing and is particularly advantageous in low-resource settings. These advantages stem from the use of a portable pH meter, in-house produced cricket cholinesterase, a single buffer solution, and room temperature reaction conditions.

Optimization of the micro-electrometric method revealed that cricket cholinesterase (ChE) exhibits significant sensitivity to the presence of OP and CM pesticides. This sensitivity was demonstrated by the ability of the method to detect variations in enzyme activity under different stock ATCh substrate concentrations and environmental conditions. Notably, the optimized conditions using a 30% w/v stock ATCh substrate concentration at a consistent temperature of 25°C provided the best balance between assay sensitivity and practical applicability. Optimization of the microelectrometric method using cricket ChE has shown significant potential for the detection of OP and CM pesticide residues. The sensitivity of ChEs to pesticides is crucial for environmental monitoring and public health. This sensitivity is primarily due to the biological role of the enzyme in hydrolyzing acetylcholine, a neurotransmitter, in which OP and CM pesticides are inhibited by phosphorylation or carbamylation, leading to neurotoxic effects [26].

This study showed that variations in enzyme activity, such as those observed with different ATCh substrate concentrations and environmental conditions, can effectively indicate the presence of these neurotoxic compounds. Using a 30% w/v stock ATCh substrate concentration at a consistent temperature of 25°C provided an optimal balance. This concentration is higher than the original 7.5% used in the Muhamadd method [20], which may be caused by the small amount of ATCh substrate initially used. This concentration was high enough to allow for measurable changes in enzyme activity while being low enough to avoid substrate saturation, which can complicate the assay results [27]. Furthermore, maintaining the assay at 25°C minimizes the thermal instability of both the enzyme and the substrate, ensuring that the observed activity changes are due to inhibitors rather than environmental fluctuations. This is similar to the reaction temperature in our previous method [13,23], compared to the 37°C of the previous studies [28,29]. The ability to conduct these tests rapidly and cost-effectively renders this approach highly beneficial, particularly in regions with limited access to sophisticated laboratory setups. Thus, developing and applying this cricket ChE-based bioassay represents a significant step forward in pesticide residue detection, offering a practical solution to global challenges in food safety and environmental health.

This study indicated that under optimal conditions, the LOD for standard pesticides was slightly lower than that for pesticide-spiked samples in both OPs and CMs. This discrepancy can be attributed to the acidity of the vegetable matrix and the solvent used in the extraction process. The study established that the LODs for OPs and CMs ranged from 0.036 to 0.086 ppm, which is within the range of the LODs for a paper-based AChE inhibition assay using commercial AChE to detect OPs, reported between 0.003 and 0.600 ppm [30], and our previous colorimetric method, which used the same cricket cholinesterase (LOD 0.002–0.508 ppm) [23]. The results were superior to those of the qualitative acetylcholinesterase detection kit, which was modified from the Ellman and ELISA methods and used honeybee heads as the source of ChE, exhibiting a LOD range of 0.50–4.80 ppm [31]. Furthermore, the potentiometric enzyme inhibition-based OP biosensor technique demonstrated a LOD of 0.038 μM and a linearity range of 50 × 10^3^ to 2.5 × 10^3^ μM for trichlorfon detection, with an incubation time of 15 min and good storage stability of 30 days [32]. This indicates that the cricket ChE can be effectively used in method development and has potential for future applications.

Comparative analysis with traditional GC-FPD methods demonstrated a high correlation, indicating that the microelectrometric method maintained its accuracy and reliability. Statistical analysis underscored the validity of the method, as evidenced by its high sensitivity, specificity, and predictive value. Low levels of pesticide residues in samples occasionally result in false negative results. However, because the method achieved over 82% efficiency for all indicators, particularly a 96% positive predictive value, it is suitable as a screening test for the detection of OP and CM residues in vegetable samples. The analysis process, which requires 20 min for the preparation of 10 samples and 40 min for the ChE inhibition method, along with the low cost of the test materials, shows that this method can serve as an effective preliminary screening tool in laboratories with limited resources. Additionally, the developed method avoids the issue of false positives caused by vegetables with intensely dark colors, which is a common problem in colorimetric methods.

The microelectrometric method developed in this study represents a significant advancement in the detection of pesticide residues. By leveraging cricket cholinesterase, this method provides a practical, low-cost, and rapid diagnostic tool that can be readily applied in diverse settings including field applications and routine monitoring by regulatory agencies. The adoption of this method can improve the monitoring of pesticide residues in agricultural products, thereby enhancing food safety and public health.
